# Difficult to treat absence seizures in children: A single-center retrospective study

**DOI:** 10.3389/fneur.2022.958369

**Published:** 2022-09-29

**Authors:** Samo Gregorčič, Jaka Hrovat, Neli Bizjak, Zvonka Rener Primec, Tadeja Hostnik, Blaž Stres, Mirjana Perković Benedik, Damjan Osredkar

**Affiliations:** ^1^Department of Pediatric Neurology, University Children's Hospital, University Medical Centre Ljubljana, Ljubljana, Slovenia; ^2^Faculty of Medicine, Centre for Developmental Neuroscience, University of Ljubljana, Ljubljana, Slovenia; ^3^Department of Animal Science, Faculty of Biotechnical, University of Ljubljana, Ljubljana, Slovenia; ^4^Department of Automation, Biocybernetics and Robotics, Jožef Stefan Institute, Ljubljana, Slovenia; ^5^Faculty of Civil and Geodetic Engineering, Institute of Sanitary Engineering, University of Ljubljana, Ljubljana, Slovenia

**Keywords:** anti-seizure medication, clinical features, first line treatment, difficult to treat absence seizures, prognostic factors, remission

## Abstract

**Objectives:**

The aim of this study was to analyse the characteristics of typical absence seizures (AS), myoclonic AS and AS with eyelid myoclonia in children and to find associations between these characteristics and difficult to treat absence seizures (DTAS).

**Methods:**

This was a single-center retrospective study. Electronic health records of pediatric patients with a clinical diagnosis of AS treated at a single tertiary epilepsy center between January 2013 and June 2020 were reviewed. Clinical characteristics, seizure information, ASM, and therapeutic response of patients were recorded. All patients were followed up for at least 1 year. DTAS were defined as failure to achieve remission after treatment with at least 2 anti-seizure medications (ASM), regardless of whether remission was achieved eventually in the study period.

**Results:**

Data from 131 patients were available for analysis. Remission was achieved after the first ASM treatment in 81 (61.8%) patients, and eventually in 120 (91.6%) during the study period. Epilepsy was classified as DTAS in 18 (13.7%) patients. AS were more often difficult to treat in patients with myoclonic AS and AS with eyelid myoclonia (40.0%), compared with patients with typical AS (11.4%; *p* = 0.012, 95% CI 1.480–25.732). A positive family history of epilepsy (*p* = 0.046; 95% CI 1.021–8.572), a higher seizure frequency (*p* = 0.023, 95% CI 1.009–1.126) prior to ASM treatment, and longer time between seizure onset and treatment onset (*p* = 0.026; 95% CI 1.006–1.099) were also associated with DTAS.

**Significance:**

Our study suggests that several clinical characteristics of AS are associated with DTAS. One of these was the time between onset of AS and initiation of ASM treatment, which can be shortened with better care, suggesting that early diagnosis and treatment may improve prognosis in pediatric patients with AS. These findings remain to be confirmed in larger prospective studies.

## Introduction

Absence seizures (AS) are sudden, relatively brief (3–30s) disturbances of consciousness associated with lack of voluntary movement and distinctive electrographic spike-wave discharges (SWD) at 2.5–4 Hz (1). They are common in children, but may persist or occur in adulthood and can be present along with other seizure types in various age-(in)dependent epilepsies. Based on clinical features and electroencephalographic (EEG) characteristics, AS can be classified into typical AS, atypical AS, myoclonic absence seizures, and absence seizures with eyelid myoclonia (2). AS represent a significant burden for pediatric epilepsy patients and their families, not only because of seizure symptoms but also because of comorbidities, including cognitive, behavioral and psychiatric impairments and academic difficulties that may persist even when seizures are eventually controlled (3–6). The outcome of pediatric patients with AS can vary: for example, children with childhood absence epilepsy (CAE) may end up with remission ≥12 months (~65–82%); persistent refractory CAE (~11%), or AS may evolve into an epilepsy syndrome in (~15%), such as juvenile myoclonic epilepsy (JME) (7).

The first-line anti-seizure medication (ASM) is effective in only about 60% of cases, with valproate (VPA), ethosuximide (ESM) and lamotrigine (LTG) being the most effective (8). Age related remission does not occur in about 10% of patients with CAE and 24% with JAE. It is also possible that the choice of first-line ASM affects treatment outcome, as ESM has been shown to have potential disease modifying effects in CAE (9). Pharmacoresistance of AS has been associated with learning difficulties, anxiety, and depression, and may generally be an obstacle in daily (social) life (10, 11). Many studies in recent years have associated various factors (age of onset, presence of generalized tonic-clonic seizures (GTCS), types of absences, EEG abnormalities, etc.) with poorer treatment response and disease prognosis, but the results are inconclusive, and definitive prognostic factors remain elusive (12). Given the high incidence of AS in childhood, there remains an unmet need for optimal treatment guidelines with ASM because of the wide range of possible outcomes and potential for various side effects (13).

The objectives of this single-center retrospective study were to (1) describe the clinical characteristics of patients with AS and the sequence of ASM therapy; (2) provide exploratory analysis of the associations between these characteristics and observed response to ASM with particular interest in characteristics associated with difficult to treat AS (DTAS).

## Methods

### Study design

In this cohort, single-center retrospective study electronic health records (EHR) of patients with a clinical diagnosis of AS who were treated at the Department of Pediatric Neurology, University Children's Hospital, University Medical Center Ljubljana, Slovenia, between January 2013 and June 2020, were evaluated. Our tertiary epilepsy center is a full member of the European Reference Network EpiCARE (https://epi-care.eu/). The study was approved by Medical Ethics Committee of the Republic of Slovenia (No. 0120-596/2019/13).

### Patient selection

All patients with onset of typical AS, myoclonic AS and AS with eyelid myoclonia before the age of 18 years were included in the study. These patients could have had AS as part of any of the following syndromes: childhood absence epilepsy (CAE), juvenile absence epilepsy (JAE), or juvenile myoclonic epilepsy (JME). The exclusion criteria were atypical AS, AS as part of any of the more severe epilepsy syndromes (i.e. Lennox-Gastaut syndrome or Dravet syndrome), a genetically confirmed syndrome (broader than epilepsy), refusal of ASM therapy, follow-up of less than 1 year, and establishment of diagnosis before June 2010, when electronic health record (HER) system was not yet introduced to our hospital.

### Diagnosis of AS

The diagnosis of absence seizures was established by a pediatric neurologist after evaluating patient's history, clinical features and patient's EEG. The classification of epilepsy into CAE with typical AS, myoclonic AS and AS with eyelid myoclonia was re-evaluated according to ILAE criteria (2) and confirmed by an experienced pediatric epileptologist (MPB).

### Data collection

Study data were collected using the in-house EHR system and pooled in the REDCap electronic data capture tools hosted at University Medical Center Ljubljana (14). Clinical features, including sex, potential risk and trigger factors for seizures, age at onset of AS, qualitative and quantitative characteristics of AS, comorbidities, among others, were collected. Seizure frequency, usual seizure duration and the occurrence of generalized tonic-clonic seizures (GTCS) at any time in the past were also noted (as reported by parents).

From the course of the disease development and treatment response, we calculated the time from AS onset to the first examination by a pediatric neurologist, establishment of the diagnosis of AS, onset of ASM treatment, remission (if applicable), and discontinuation of ASM treatment (if applicable).

### ASM treatment and remission

The ASM of first choice and all subsequent changes in ASM (where applicable) were noted. For each ASM or combination of ASM, adverse drug reactions and/or the reason for substitution, if occurred, were noted. The designation of effective ASM treatment was given to a particular ASM if remission was achieved once it was introduced. Remission was defined as complete seizure freedom for at least 3 months which lasted until the end of the study period; if the seizures relapsed after a certain period and persisted to the end of the observed period, we have not considered the child to achieve remission in the study period. Only patients who remained in remission after discontinuation of ASM until the end of the study period were classified as being able to discontinue ASM.

### Difficult to treat AS and non-responsive AS

AS were classified as difficult to treat if remission was not achieved after at least two appropriate ASM treatments at the recommended daily dosages, thus being pharmacoresistant at some point in the study period, according to definition by ILAE (15). Some patients with DTAS still achieved remission eventually within the study period with further ASM modifications. In patients who were treated with at least two appropriate ASM but never achieved remission within the study period, AS were classified as non-responsive AS.

### Statistical analysis

The Pearson χ^2^ test and Fisher's exact test were performed to compare categorical data. Linear regression was used to assess the association between the time from onset of AS to onset of treatment and the time from onset of treatment to remission, or time of discontinuation of ASM; and to assess the association between the time from treatment onset to remission and the time of discontinuation of ASM. All time periods were calculated in months. To assess which characteristics are associated with DTAS, a binary logistic regression was completed, with the Hosmer-Lemeshow test to test goodness of fit, given as *p-*value and a 95% CI of Exp (B). Statistical analysis was performed using IBM SPSS Statistics version 27 (IBM Corporation, Armonk, NY, USA). A value of *p* < 0.05 was considered as statistically significant.

## Results

### Patient selection process

A total of 229 patients who met the study inclusion criteria were initially identified. Ninety-eight patients were excluded according to the exclusion criteria (the flow of patient inclusion/exclusion is presented in Supplementary Figure 1). The final number of patients included in the analysis was 131.

### Clinical characteristics

The basic clinical data of children with AS enrolled in the study are provided in Table 1. All patients were evaluated at least twice by a pediatric neurologist and all patients had at least one EEG recorded prior to ASM onset and one after reaching full dose of the prescribed ASM. The median follow-up time (from the first evaluation by a pediatric neurologist to the end of the study period, June 2019) was 4.2 years (range, 1.0–13.4 years).

**Table 1:** Baseline and clinical characteristics of the subjects.

	**Number of subjects (%)**
Enrolled patients	131 (100)
**Sex**	
Male	55 (42.0)
Female	76 (58.0)
**Age (years)**	
1–4	19 (14.5)
5–8	74 (56.5)
9–12	25 (19.0)
13+	13 (10.0)
**Potential risk factors for epilepsy**	
Family history of epilepsy	27 (20.6)
Personal history of febrile convulsions	20 (15.3)
Complications during labor and delivery	22 (16.8)
**Co-occurring disorders**	
Attention deficit/hyperactivity disorder	11 (8.4)
Developmental disorders	7 (5.3)
**Type of AS**	
Typical AS	122 (93.1)
Myoclonic AS/absences with eyelid myoclonia	9 (6.9)
Occurrence of GTCS	19 (14.5)
**Average seizure frequency per day**	
<1	21 (16.0)
1–10	95 (72.5)
11–20	8 (6.1)
21–30	6 (4.6)
>30	1 (0.8)
**Duration of seizures**	Time (s)
Median (range)	5 (2–60)
**Difficult to treat AS**	18 (13.7)
**Non-responsive AS**	11(8.4)

Difficult to treat AS: failure to achieve remission after treatment with at least 2 ASM, regardless of whether remission was achieved eventually in the study period; non-responsive AS: failure to achieve remission after at least 2 ASM throughout the study period.

### Seizures and EEG characteristics

The median age at the onset of AS was 6.2 years (range, 1.3–17.0; age and sex distribution of patients at AS onset is shown in Supplementary Figure 2). The AS were first noticed by parents in 107 cases (81.7%), by patients themselves in 8 cases (6.1%), by teachers in 6 cases (4.6%), by physicians in 3 cases (2.3%), and unknown in remaining seven cases (5.3%). The median duration from the seizure onset to the establishment of diagnosis was 4.0 months (range, 0–55.0 months); some patients reported the presence of AS for years prior to the first evaluation by a pediatric neurologist, the longest reported time period being 55 months.

In 122/131 patients (93.1%), AS were classified as typical, while the remaining nine patients (6.9%) had AS with special features (myoclonic AS and AS with eyelid myoclonia). Both seizure duration and frequency varied greatly among subjects. The median number of reported seizure frequency was 3 per day (range, 0–100). The median seizure duration was 5s (range, 2–60 s). The positive history for GTCS was present in 19 patients (14.5%).

All patients had at least one EEG performed prior to ASM onset. In all but five patients (3.8%) typical SWD of approximately 3Hz were present on EEG, thus confirming the diagnosis of AS; in the five patients EEG was inconclusive, but clinical features were in alignment with typical AS in these patients. In 82 patients (62.6%) the epileptiform activity was pronounced over the frontal regions. In 116 patients (88.5%) hyperventilation performed at the time of EEG recording provoked AS, and 37 (28.2%) patients had photoparoxysmal response on intermittent photo stimulation. Parents reported that tiredness (24 patients; 18.3%), stress (17 patients; 13.0%), and physical activity (two patients; 1.5%) also could provoke AS.

### Treatment

The median time from seizure onset to onset of treatment was 4.0 months (range, 0–55 months). Various ASM were used as initial monotherapy; subsequent ASM could have been used as monotherapy or a in combination (Supplementary Table 1). Valproic acid was the most commonly used first line ASM as it was used in 95 patients (72.5%), followed by ethosuximide (21 patients, 16.0%), levetiracetam (12 patients, 9.2%), lamotrigine, clobazam and cannabidiol (one patient each, 0.8%). One patient received cannabidiol as the first line treatment because the parents refused standard ASM and would prefer not to treat the child if this option was not available; the number of seizures decreased after treatment with cannabidiol, but remission was not achieved in the study period.

**Figure 1:**
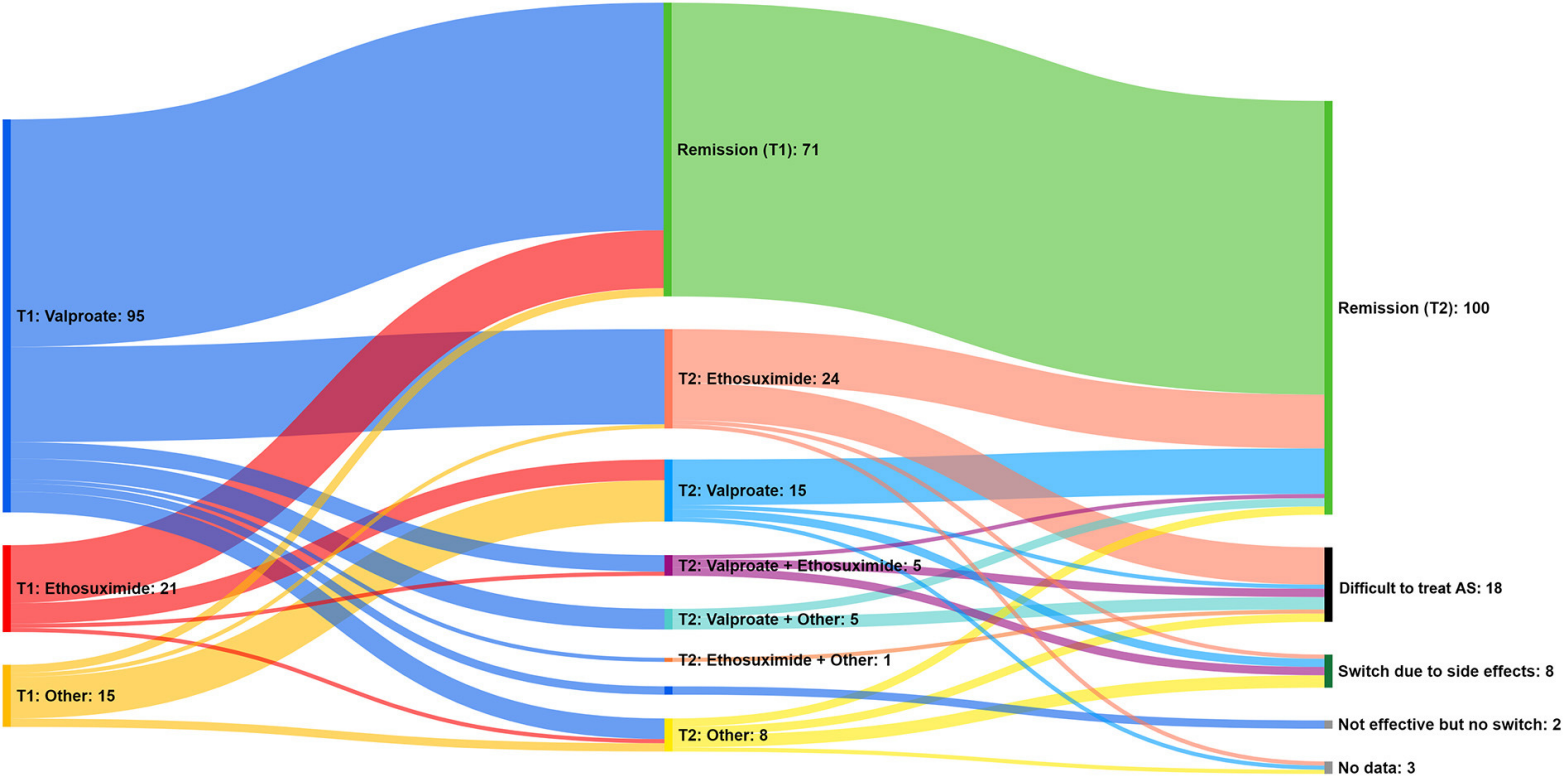
The flow of patients regarding 1st and 2nd line ASM treatment and the observed outcomes. Only patients who did not switch to next ASM treatment for any reason (such as side effects) were considered to have achieved remission for the purpose of this figure.

Remission was achieved after the first ASM in 81/131 (61.8%) patients, and additional 25 patients (19.1%) reported reduced seizure frequency. Remission was eventually achieved in 120 (91.6%) patients after modifying ASM treatment within the study period. The median time from onset of treatment with ASM to the onset of remission was 1.7 months (range, 0–75.3 months). For the remaining 11 (8.4%) patients, the ongoing treatment was not effective by the end of the observed study period. Figure 1 shows the flow of patients regarding 1st and 2nd line ASM treatment in relation to the observed outcomes.

**Figure 2:**
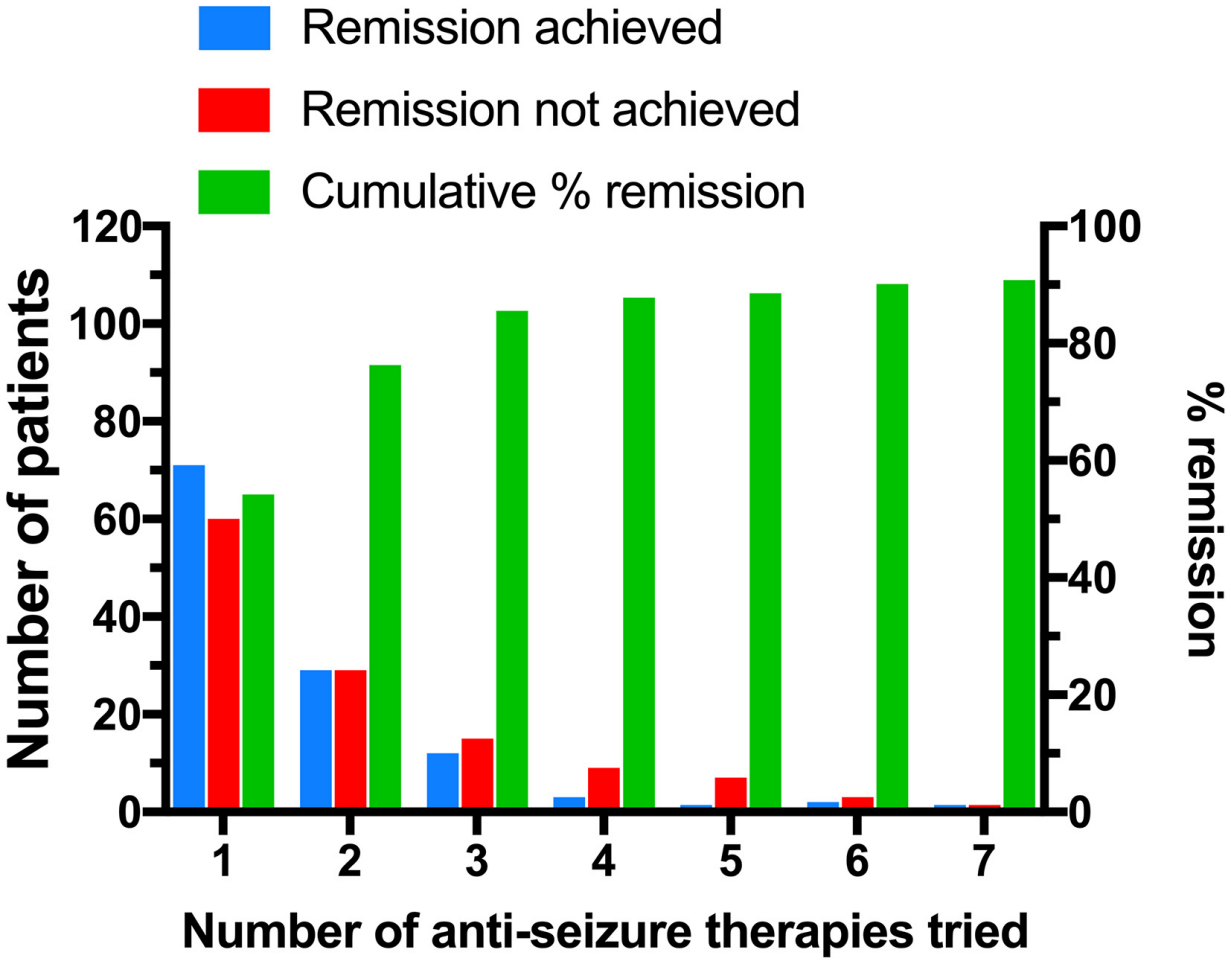
Success of ASM therapy on achieving remission. Blue: patients who have achieved remission on particular ASM therapy (monotherapy or combination therapy). Only patients who did not switch to next ASM treatment for any reason (such as side effects) were considered to have achieved remission for the purpose of this figure. Red: patients who have not achieved remission on particular ASM therapy. Green: cumulative percentage of patients achieving remission (right y axis).

Figure 2 presents the data on effectiveness of the first line and subsequent ASM treatments (when applicable) with regards to achieving remission. Of all 131 patients, 58 patients (44.3%) switched to a second line therapy; 48 patients (36.6%) switched because the first-line therapy was not effective; the remaining 10 patients (10.6%) switched due to side effects, in spite of the first line therapy being effective in achieving remission. Two patients remained on the first line therapy even if being ineffective because of their parents' reasoning. The second therapy was further substituted in 27 (20.6%) cases, while 12 patients (9.2%) were receiving four or more different therapies as monotherapy or in combination, of which two patients (1.5%) received as much as seven ASM, which was the maximum number. Of all patients, 100 patients (76.3%) achieved remission after the first line or second ASM treatment and did not switch to the 3rd ASM for any reason. Four patients discontinued treatment on their own behalf; two patients remained in remission, and two patients continued to have seizures.

After achieving remission, treatment was successfully discontinued in 50 (38.2%) patients in the study period. In those patients, the median length of the time period between seizure onset to discontinuation of treatment with ASM was 41.7 months (range, 13.3–75.1 months); from treatment onset to discontinuation of treatment with ASM was 34.5 months (range, 1.2–74.6 months); and from the onset of remission to discontinuation of treatment with ASM was 33.8 months (range, 1.2–57.9 months). Only one patient discontinued ASM treatment in the first year: this patient had CAE with typical 3 Hz spike-wave pattern on EEG and received treatment with VPA, but the parents have decided to discontinue the treatment after 1.2 months on their own accord. A follow-up EEG after 5 months later did not show the SWD anymore and the boy did not have clinical signs of AS.

The results of our analysis have shown a statistically significant difference in the effectiveness of the three most often used first-line ASM, namely VPA, ESM and levetiracetam, at achieving seizure remission (*p* = 0.014). The results are presented in Table 2.

**Table 2:** Effectiveness of first-line ASM treatment.

**ASM**	**N**	**Treatment effectiveness (%)**	**χ^2^**	**df**	***p*-value**
Valproic acid	95	63 (66.3)			
Ethosuximide	21	15 (71.4)	8.55	2	0.014
Levetiracetam	12	3 (25.0)			

χ^2^, value of chi-square test statistic; df, degrees of freedom.

Further analysis suggests that the effectiveness of levetiracetam in achieving seizure control was inferior compared to VPA (*p* = 0.010) or ESM (*p* = 0.014). However, there was no significant difference between effectiveness of VPA and ESM (*p* = 0.799).

### Associations between clinical characteristics and DTAS

Epilepsy was classified as DTAS in 18 (13.7%) patients, however 7/18 of these patients still eventually achieved remission in the study period, while 11/18 never responded to ASM. Logistic regression has suggested a higher number of patients with DTAS among patients with myoclonic AS and AS with eyelid myoclonia (40.0%), compared to patients with typical AS (11.4%; *p* = 0.012, 95% CI 1.480–25.732). Positive family history of epilepsy was also found to be significantly related to DTAS (*p* = 0.046; 95% CI 1.021–8.572). A higher seizure frequency has been related to DTAS (*p* = 0.023, 95% CI 1.009–1.126), but not the duration of seizures (*p* = 0.549).

A longer time from the seizure onset to treatment onset was significantly related to DTAS (*p* = 0.026; 95% CI 1.006–1.099). Age of onset of AS was not significantly related to DTAS (*p* = 0.179), as well as presence of GTCS (*p* = 0.779), frontal predominance of epileptiform discharges on EEG (*p* = 0.062) or other forms of focal activity (*p* = 0.324), or which ASM was chosen as the first line of treatment (*p* = 0.403).

### Associations between clinical characteristics and discontinuation of ASM treatment

In children who did not have DTAS, the time from onset of AS to onset of treatment with ASM was not significantly related to time from onset of treatment to remission (*p* = 0.390) or time from onset of treatment to discontinuation of treatment (*p* = 0.151). Time from beginning of treatment to remission was not associated with the time from beginning of treatment to discontinuation of treatment (*p* = 0.056).

## Discussion

Although there is strong evidence supporting the choice and efficacy of first-line ASM treatment of AS, approximately 20% of patients with AS do not respond favorably to ASM treatment (16). In our study, 8.4% of patients with AS did not achieve remission within the study period. Our data confirm the findings of other authors that patients with myoclonic AS and AS with eyelid myoclonia are more often difficult to treat, compared with patients with typical AS (12). What our study also suggests is that a longer time interval between the onset of AS and the initiation of treatment, a higher pre-treatment seizure frequency, and a positive family history of epilepsy were also found to be associated with DTAS.

In CAE, the reported response rate to ASM varies from 56 to 95%, depending on the definition of the CAE population studied, the length of the observation period, and the definition/measurement of outcomes (9, 17, 18). In JAE, seizure freedom can be achieved in 62–84% with ASM (19). In our study, remission was achieved after the first ASM in 61.8% and eventually in 91.6% of patients at a median follow-up of 4.3 years, whereas 8.4% of patients with AS did not achieve remission within the study period. Our results are consistent with the findings of others.

A Cochrane review by Brigo and Igwe compared the efficacy of ESM, VPA, or LTG as first-line therapy for AS in children and adolescents (20). The study suggests that the optimal initial monotherapy is ESM, or if AS and generalized tonic-clonic seizures coexist, VPA should be preferred. Although it is generally believed that ASM can treat AS without affecting the long-term prognosis for remission, there is some evidence that initial treatment with ESM is more likely to result in remission than initial treatment with VPA (9). ESM and VPA were found to be the most effective first-line ASM treatment in our study (71 and 63%, respectively), representing a better response than in the study by Berg et al. (9) (56 and 59%, respectively). VPA and ESM were superior to levetiracetam in achieving seizure remission, whereas no significant difference in effectiveness was found between VPA and ESM. In addition, no association was found between choice of the first-line ASM and DTAS.

The results of our study suggest an association between a longer time from the onset of AS and the initiation of treatment, a higher seizure frequency, and a positive family history of epilepsy with DTAS. Both longer lead time and higher seizure frequency predispose patients to a higher seizure burden before treatment initiation. Patients who have many seizures before treatment or who have an inadequate response to initial treatment with ASM are more likely to have refractory epilepsy (21, 22). The association between a high number of seizures before treatment and later intractability has been studied in the context of the experimental phenomenon of kindling (23). Some evidence suggests that early treatment may alter the natural course of epilepsy (24). The time between seizure onset to treatment initiation can be shortened with better care, underscoring the importance of early diagnosis and appropriate treatment for optimal patient outcome. Based on our results, it is possible to speculate that early treatment of AS plays a more important role in preventing the development of DTAS than the choice of first-line drug itself (when choosing between ESM, VPA or levetiracetam), but this would need to be further investigated in studies specifically designed for this purpose.

### Limitations

Our study has limitations. The retrospective and observational nature of the study did not allow systematic collection of data resulting in missing data and lower statistical power. The number of patients with myoclonic AS or AS with eyelid myoclonia in our study was low compared to patients with typical AS, which may affect the interpretation of data. Also, the number of patients with DTAS was low compared to patients who responded to ASM treatment, resulting in the danger of overfitting the data. The study design also did not allow us to investigate the associations found between clinical parameters and observed outcomes in detail and precluding identification of causative relationships. Genetic data was not available for the study, although a positive family history of epilepsy was associated with DTAS.

## Conclusions

The results of our study suggest that a positive family history of epilepsy and certain types of AS epilepsy (myoclonic AS and AS with eyelid myoclonia) are more commonly associated with DTAS. Early diagnosis and treatment with an appropriate first-line ASM may improve the response to ASM treatment in pediatric patients with AS. Because it is possible to shorten time to treatment in certain clinical situations, this should be considered an important principle in treating pediatric patients with AS to improve their outcomes. These results need to be confirmed in larger and prospective studies.

## Data availability statement

The original contributions presented in the study are included in the article/Supplementary material, further inquiries can be directed to the corresponding authors.

## Ethics statement

The studies involving human participants were reviewed and approved by Medical Ethics Committee of the Republic of Slovenia. Written informed consent from the participants' legal guardian/next of kin was not required to participate in this study in accordance with the national legislation and the institutional requirements.

## Author contributions

SG, JH, MP, and DO conceived and designed the analysis. SG, JH, and MP collected the data. NB, ZR, TH, and BS contributed data. SG, JH, BS, and DO performed the analysis. SG, JH, ZR, MP, and DO wrote the paper. All authors contributed to the article and approved the submitted version.

## Conflict of interest

The authors declare that the research was conducted in the absence of any commercial or financial relationships that could be construed as a potential conflict of interest.

## Publisher's note

All claims expressed in this article are solely those of the authors and do not necessarily represent those of their affiliated organizations, or those of the publisher, the editors and the reviewers. Any product that may be evaluated in this article, or claim that may be made by its manufacturer, is not guaranteed or endorsed by the publisher.

## Supplementary material

Supplementary Figure 1 Flow chart of patient selection for the study.

Supplementary Figure 2 Age distribution of patients at absence seizure onset, stratified by gender.

Supplementary Table 1 Number (percentage) of subjects receiving a particular ASM at any time in the study period and percentage of reported adverse effects for a particular ASM.

## Abbreviations

AS: absence seizures
ASM: anti-seizure medication
CAE: childhood absence epilepsy
DTAS: difficult to treat absence seizures
EEG: electroencephalogram/electroencephalography
EHR: electronic health record
ESM: ethosuximide
JAE: juvenile absence epilepsy
JME: juvenile myoclonic epilepsy
LTG: lamotrigine
SWD: spike-wave discharges
VPA: valproate.
