# Supplementary figures and images for: Difficult to treat absence seizures in children: A single-center retrospective study
Source: Front Neurol. 2022 Sep 29;13:958369. doi: 10.3389/fneur.2022.958369 (PMC9556893; doi:10.3389/fneur.2022.958369)

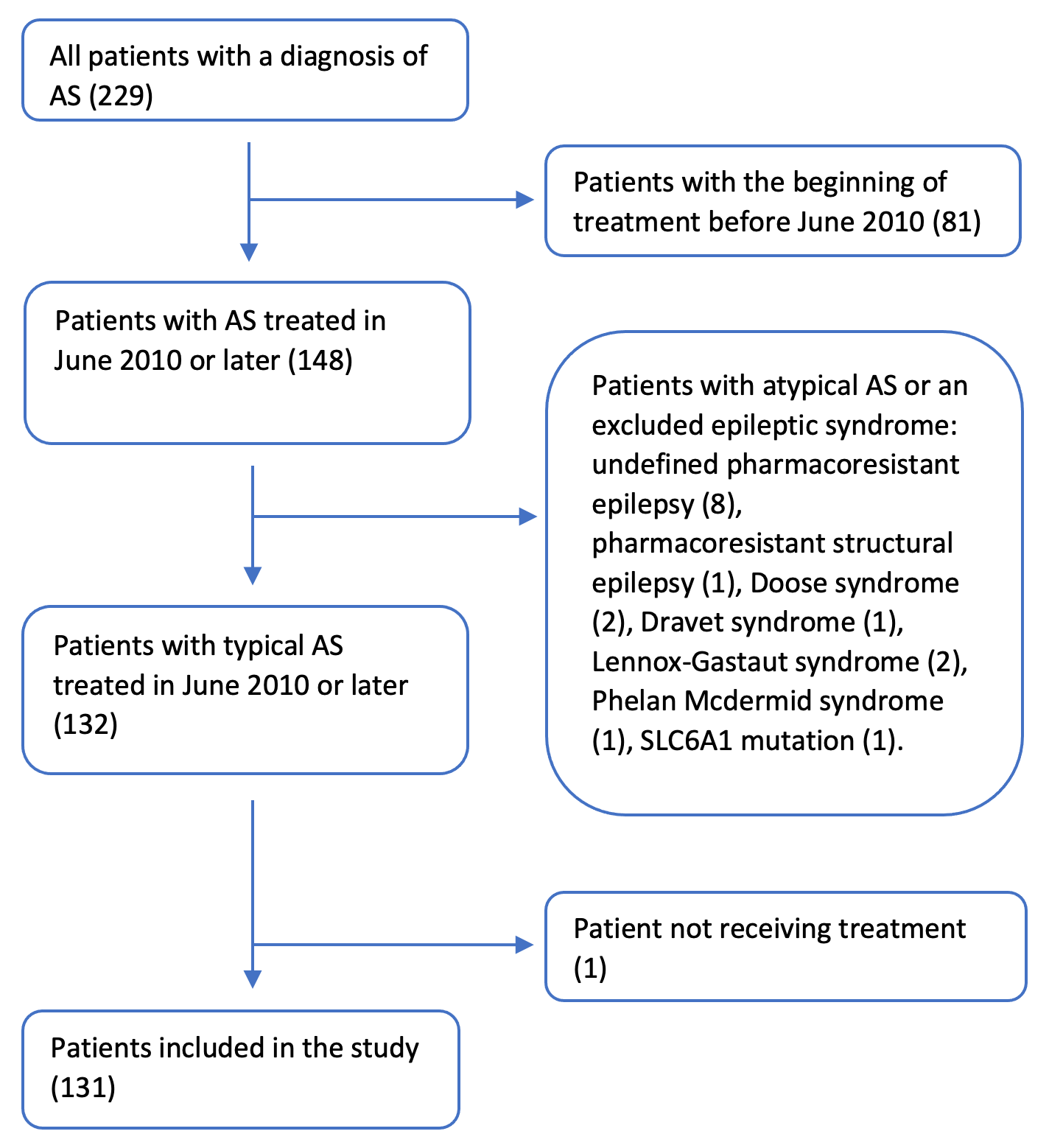

Supplement: Supplementary Figure 1 — Flow chart of patient selection for the study. [file Image_1.TIFF]

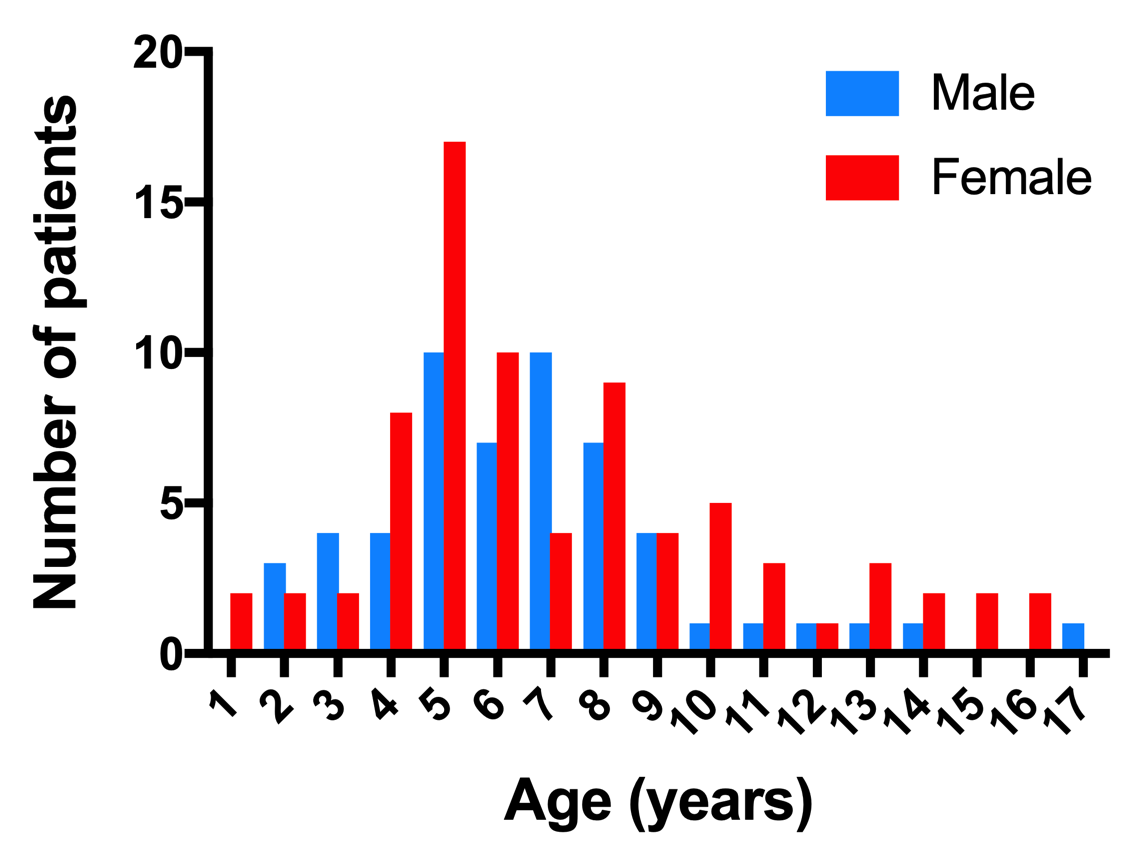

Supplement: Supplementary Figure 2 — Age distribution of patients at absence seizure onset, stratified by gender. [file Image_2.TIFF]
