# Supplementary material for: Difficult to treat absence seizures in children: A single-center retrospective study
Source: Front Neurol. 2022 Sep 29;13:958369. doi: 10.3389/fneur.2022.958369 (PMC9556893; doi:10.3389/fneur.2022.958369)
Supplement: Supplementary Table 1 — Number (percentage) of subjects receiving a particular ASM at any time in the study period and percentage of reported adverse effects for a particular ASM. [file Table_1.DOCX]

|  | Number of subjects (%) | |
| --- | --- | --- |
| ASM | Receiving | Adverse effects (*) |
| Valproate | 114 (87.0) | 42 (36.8) |
| Ethosuximide | 56 (42.7) | 21 (37.5) |
| Levetiracetam | 20 (15.3) | 9 (45.0) |
| Lamotrigine | 16 (12.2) | 3 (18.8) |
| Clobazam | 9 (6.9) | 4 (44.4) |
| Zonisamide | 6 (4.6) | 0 (0.0) |
| Clonazepam | 5 (3.8) | 2 (40.0) |
| Cannabidiol | 5 (3.8) | 0 (0.0) |
| Topiramate | 2 (1.5) | 1 (50.0) |
| Sultiame | 1 (0.8) | 0 (0.0) |

*Percentage of patients with side effects receiving a certain ASM.
